# Bioorthogonal azido-S1P works as substrate for S1PR1

**DOI:** 10.1016/j.jlr.2022.100311

**Published:** 2022-11-10

**Authors:** Christine Sternstein, Jan Schlegel, Markus Sauer, Jürgen Seibel

**Affiliations:** 1 Institute of Organic Chemistry, Julius-Maximilians-Universität Würzburg, Würzburg, Germany; 2 Department of Biotechnology and Biophysics, Biocenter, Julius-Maximilians-Universität Würzburg, Würzburg, Germany

**Figure:**
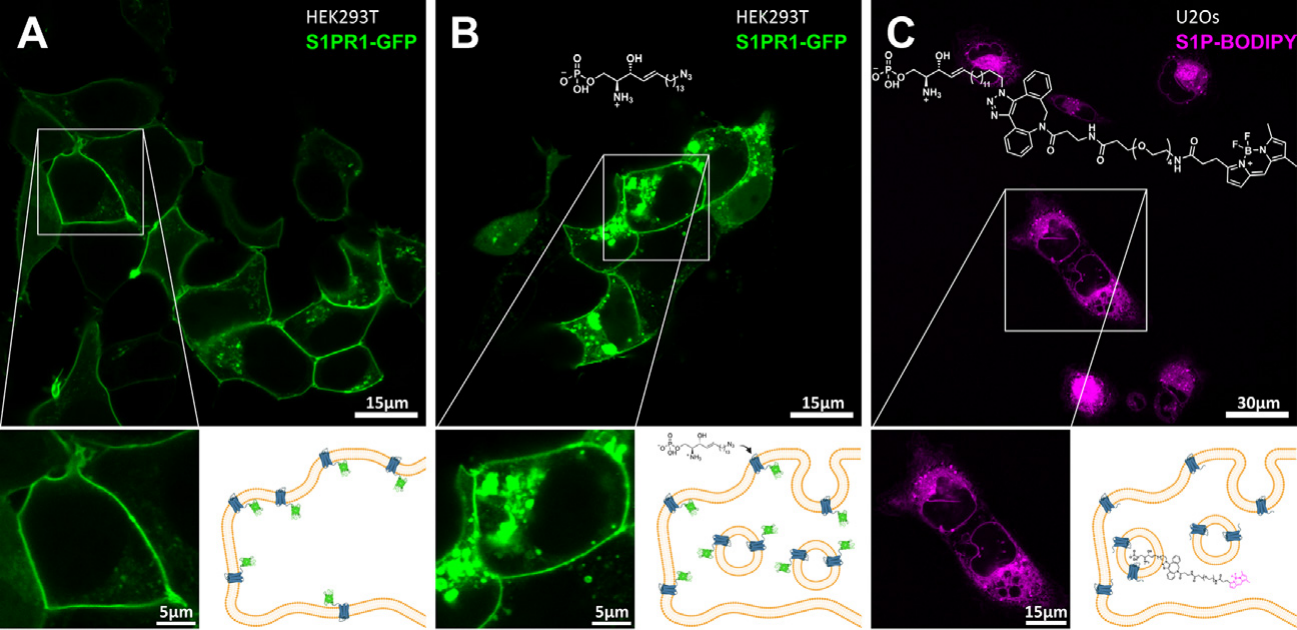

Sphingosine-1-phosphate (S1P) and its G protein-coupled receptors S1PR1-5 play an important role in cellular processes and are associated with many diseases (1, 2). For that reason, the investigation of the S1P metabolism and its intracellular tracking is of high scientific interest. Azido-functionalized sphingolipids are tolerated by biological systems and can be modified via click chemistry with alkyne-substituted molecules (3). We synthesized a clickable S1P derivative with a terminal azido function (S1P-N_3_) in 11 steps combining two synthetic approaches. Following the protocol by Lang *et al.* (4) yielded *N*-Boc-protected azido-sphingosine, followed by phosphorylation of the primary alcohol to the target molecule. To investigate if S1P-N_3_ works as a substrate for S1PR1, living human embryonic kidney 293T (HEK293T) cells, showing very low transcription of S1PRs, were transfected with S1PR1-GFP and incubated with S1P-N_3_. Prior to the addition of the lipid, the receptor was localized within the plasma membrane (A). After feeding with S1P-N_3_, a considerable amount of the receptor was detected within the cell (B). This suggests the activation and consecutive internalization of S1PR1 by our modified substrate. These findings strongly indicate that S1P-N_3_ is still functional, making it a powerful tool for studying the S1P metabolism (3). The applicability for this was confirmed by incubating U2Os cells, showing adequate endogenous levels of S1PR3 and S1PR5, with S1P-N_3._ Staining by click reaction with a DBCO-BODIPY dye and its visualization within the cells by confocal microscopy showed that the S1P-dye conjugate and its metabolites were mainly localized intracellularly at the nuclear membrane and in the endoplasmic reticulum (C). Until now, colabeling experiments of the clicked S1P-N_3_ within the S1PR1-GFP construct were not successful. As the alkyl chain of S1P is known to interact with the intracellular region of the S1PR1-binding pocket, the terminal azido-function is probably buried and not accessible for the click reaction (1). We demonstrated that S1PR1-GFP of HEK293T cells accepts S1P-N_3_ as a substrate and that S1P-N_3_ and its metabolites can be visualized and localized within U2Os cells.

**EQUIPMENT AND METHODS:** HEK293T cells were seeded into 8-well chambered cover glass (Cellvis) and transfected with S1PR1-GFP (kindly provided by Prof Meyer zu Heringdorf) using PEI 25K (Polysciences). After 24 h, cells were imaged in the presence or absence of 1 μM S1P-N_3_. To visualize subcellular localization of S1P-N_3_, U2Os cells were incubated for 40 min at 37°C and 5% CO_2_ with 1 μM S1P-N_3_ and clicked with 1 μM BODIPY-FL-PEG_4_-DBCO (Jena Bioscience) for 20 min. Both fluorophores, GFP and BODIPY, were imaged using a Zeiss LSM 700 AxioObserver microscope equipped with Plan-Apochromat 63×/1.4 Oil M27 objective and 488 nm laser light. Schematic illustrations were created with Biorender.com.

## Conflict of interest

The authors declare that they have no conflicts of interest with the contents of this article.
